# Inter-effort hypoxia recovery during high-intensity intermittent exercise enhances oxygen uptake at the onset of efforts while maintaining exercise tolerance

**DOI:** 10.1007/s00421-026-06176-y

**Published:** 2026-03-14

**Authors:** M. S. Norberto, G. M. Putti, T. R. Figueira, C. D. de Carvalho, F. M. Rasteiro, A. B. Marostegan, F. B. Manchado-Gobatto, M. Papoti

**Affiliations:** 1 Ribeirão Preto Medical School, University of São Paulo, Ribeirão Preto, São Paulo, Brazil; 2 Hypoxia, Sport and Health Team, School of Applied Sciences, Limeira, São Paulo Brazil; 3 School of Physical Education and Sport of Ribeirão Preto, University of São Paulo, Ribeirão Preto, São Paulo Brazil; 4 School of Applied Sciences, University of Campinas, Limeira, São Paulo Brazil

**Keywords:** Intermittent hypoxia, High-intensity interval training, Muscle oxygenation, Oxygen consumption kinects

## Abstract

Exercise training in hypoxia enhances physiological adaptations improving exercise performance. However, acute hypoxia generally reduces high-intensity exercise tolerance, limiting its application in sports training. Here, we investigated whether oxygen consumption and exercise tolerance are affected during a session of an emerging high-intensity intermittent training (HIIT) model in hypoxia. This model involves efforts in normoxia with inter-effort hypoxia (IEH) recoveries. Young active males were recruited and completed a graded exercise test in normoxia, followed by HIIT sessions under three conditions: normoxia, continuous normobaric hypoxia (FIO_2_: ~0.13), and IEH recovery, in different days and random order. Oxygen consumption, ventilatory variables and muscle oxygenation in the vastus laterali were assessed during HIIT sessions consisting of ten 1-min efforts (at 120% of maximal treadmill running speed from the graded test), with 2-min passive recoveries. Compared to normoxia, IEH recovery caused significant hemoglobin desaturation (between 95% and 88%) and a ~ 14% decrease in V̇O_2_ during recoveries. During efforts, particularly in the first 30 s, VO_2_ was significantly increased by approximately 7% in the IEH condition compared to normoxia. Notably, exercise task completion was nearly identical between normoxia (87 ± 24%) and IEH recovery conditions (87 ± 18%), but significantly lower in continuous hypoxia (44 ± 27%), along with impaired indexes of O_2_ metabolism. Additionally, IEH recovery resulted in a significantly lower pulmonary O_2_ diffusion gradient at a given V̇O_2_, suggesting a compensatory increase in blood flow. In conclusion, IEH recovery preserved muscle oxygenation and exercise performance while enhancing V̇O_2_ during efforts.

**Key-points**

- The application of hypoxia exclusively during recovery periods (IEH) resulted in higher V̇O_2_ during HIIT efforts, particularly within the first 30 s of each sprint.
- IEH recovery attenuated muscle deoxygenation and perceived exertion during the efforts compared to continuous hypoxia, suggesting improved oxygen delivery and redistribution.
- VO_2_ kinects demonstrated a faster response at the onset of sprints under IEH, suggesting a hypoxia-induced priming effect on oxidative metabolism during recovery.

## Introduction

The interaction between environmental hypoxia and physical exercise has been widely studied over the past decades. The rationale for using environmental hypoxia — whether hypobaric or normobaric — during exercise stems from its potential to induce systemic hypoxia and trigger downstream signaling effects in various organs (Burtscher et al. 2022; Faiss et al. 2024). In this context, it is well known that the activation of hypoxia-inducible factors may stimulate erythropoietin secretion and the respective erythropoiesis (Płoszczyca et al. 2018; Fernández-Lázaro et al. 2022), induce a glycolytic transcription pattern (Soo et al. 2023), improve muscle H^+^ buffering capacity (Gore et al. 2007), enhance muscle angiogenesis (Lemieux and Birot 2021), and promote nitric oxide-mediated vasodilation (Meng et al. 2019; Burtscher et al. 2022). Notably, compensatory vasodilation, increased muscle capillarization, and higher erythrocyte/hemoglobin mass (i.e., the O_2_ carrying capacity of the blood) are components of the O_2_ transport system that may be improved with repeated exposures to hypoxia (Meng et al. 2019; Lemieux and Birot 2021; Wojan et al. 2021; Burtscher et al. 2022). Ultimately, these adaptations may benefit metabolic function and exercise performance (Cerda-Kohler et al. 2022; Dragos et al. 2022). However, acute exposure to hypoxic air impairs tolerance to high-intensity exercise (Brocherie et al. 2016; Girard et al. 2017, 2020). Since achieving a minimal amount of workload at high intensities is necessary to improve physical performance (Roels et al. 2005; Millet et al. 2010; Brocherie et al. 2016), hypoxia may also have a detrimental effect on exercise adaptations and acute performance.

When incorporating hypoxia into a chronic training program, it is important to consider the acute physiological responses and the exercise tolerance to each session. In hypoxic high-intensity exercise, the work-to-rest ratio is a key determinant of whether hypoxia compromises or preserves performance (Buchheit and Laursen 2013; Girard et al. 2017). In this context, the relatively long recovery periods typical of high-intensity interval exercise allow partial restoration of O_2_ transport, which may influence the V̇O_2_ response when exercise resumes (Girard et al. 2017). Researchers have explored high-intensity exercise protocols in which hypoxia exposure does not compromise exercise tolerance, particularly through intermittent exercise protocols (Papoti et al. 2023; Li et al. 2024). One studied protocol involved 30-s all-out efforts under hypoxia, with 4-min recovery periods in normoxia, allowing participants to sustain the same mean intensity over four repetitions compared to the normoxia control condition (Takei et al. 2024). Conversely, when hypoxia is present during both effort and recovery phases, exercise performance may decline, even with very short effort durations (e.g., 5-s) (Brocherie et al. 2016).

Recently, a novel intermittent exercise protocol involving recovery periods in hypoxia but efforts in normoxia, termed inter-effort hypoxia (IEH), has yielded promising results (Papoti et al. 2023). Roels et al. (2005) implemented IEH recovery sessions in a training program for cyclists and found that maximal oxygen uptake (V̇O_2max_) increased only in the IEH group, compared to the normoxia control group. We previously reported that a high-intensity intermittent training program incorporating IEH recovery (with an inspired oxygen fraction [FIO_2_] of approximately 0.13) was superior to a normoxia control condition in improving time-to-exhaustion at 120% of maximal treadmill running velocity achieved during the graded exercise test (V_MAX_) and anaerobic capacity in amateur runners (Putti et al. 2024). Notably, independent of exercise, an session of intermittent hypoxia (i.e., inducing repeated shifts in FIO_2_) appears capable of eliciting physiological stress and adaptations (Morton and Cable 2005; Wojan et al. 2021).

To our knowledge, only a few studies have evaluated acute physiological responses to high-intensity intermittent training sessions under the IEH recovery condition (de Carvalho et al. 2023; Foresti et al. 2023; Li et al. 2024). These studies employed protocols consisting of 10 × 1-minute efforts at either 120% or 100% of V_MAX_, with 2-min of IEH recoveries at a FIO_2_ of ~ 0.13 (Foresti et al. 2023). In both studies, SpO_2_ decreased to approximately 85% during the recovery periods in the IEH condition; however, this reduction did not impair exercise tolerance, as reflected by similar rate of perceived exertion (RPE) values compared with normoxia (Foresti et al. 2023).

Given that convective O_2_ delivery and peripheral extraction of O_2_ retain functional reserves during resting and low-intensity exercise (Calbet et al. 2003), it is plausible that aerobic metabolism can be maintained despite the peripheral oxygenation (SpO_2_) reduction induced by IEH recovery (Foresti et al. 2023). Although hypoxia during exercise involving large muscle mass decreases muscle O_2_ delivery and performance (Chacaroun et al. 2019), recovery in hypoxia may elicit compensatory vasodilation and microvascular adjustments. When the condition abruptly returns to normoxia at the onset of the next effort, these transient hemodynamic adjustments may facilitate a rapid increase in O_2_ delivery and extraction, thereby mitigating the decline in muscle oxygenation typically observed during high-intensity efforts in hypoxia (Liu et al. 2017; Willis et al. 2017) and preserving performance during the exercise session.

Thus, in this study, we aimed to test the hypotheses that (1) V̇O_2_ during intermittent exercise would increase in response to IEH recovery and (2) exercise tolerance would not be impaired in this condition compared to normoxia. Additionally, a hypoxia condition during both the effort and recovery phases was included as a positive control to assess the suppressive effects of hypoxia on oxidative metabolism and exercise tolerance. We also hypothesized that IEH recovery would enhance muscle oxygenation status compared to hypoxia during the effort phases of a HIIT session.

## Methods

### Participants

Twelve recreational runners (age of 24 ± 5 years old; body mass of 74.1 ± 14.5 kg; height of 174.5 ± 8.9 cm; V̇O_2peak_ of 49.8 ± 5.3 mL/kg/min) who had not experienced any injuries or altitude exposure in the past six months participated in the study only after signing the informed consent form. Eligibility criteria included being over 18 years of age and physically active without cardiovascular disease. Exclusion criteria comprised missing one or more training sessions, presented an injury during the intervention, or being exposed to altitude outside the context of the experimental sessions. Participants were recruited without sex-based inclusion or exclusion criteria; however, the final sample comprised only male volunteers due to recruitment availability during the enrollment period. This study was approved by the university’s ethics committee and is in accordance with the Declaration of Helsinki.

### Experimental design

**Fig. 1:**
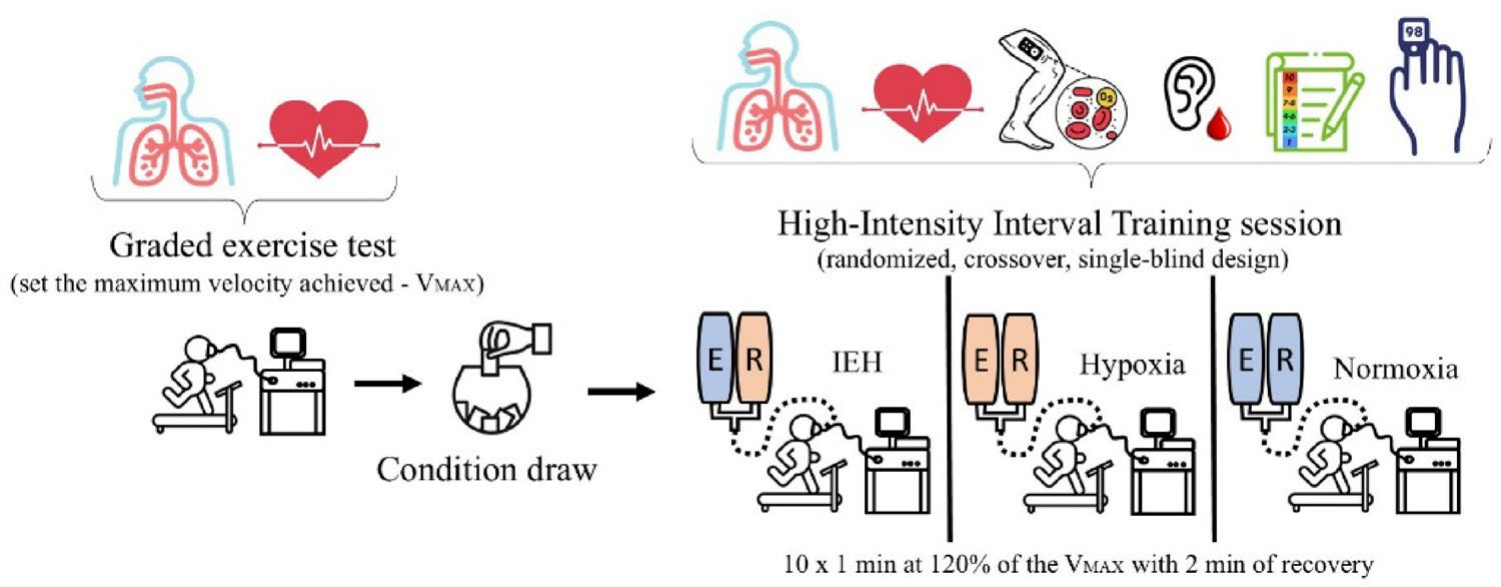
A scheme of the experimental design. This study was randomized, crossover, and single-blind, over four visits (≥48 h apart) to the laboratory. Visit 1 included a graded exercise test to exhaustion with cardiorespiratory analysis (performed in normoxia). Visits 2–4 consisted of HIIT sessions performed under different conditions: full normoxia, full hypoxic (FIO_2_ = 0.13; ~3,600 m), and efforts in normoxia with recovery in hypoxia (Inter-effort hypoxic recovery – IEH). During these HIIT sessions, pulmonary gas exchange, heart rate, and vastus lateralis muscle oxygenation were monitored; during recovery intervals only, blood lactate, rate of perceived exertion, and peripheral oxygenation (SpO_2_) were recorded

The study employed a randomized, crossover, single-blind design conducted over four visits separated by at least 48 h, as illustrated in Fig. 1. In the first visit, participants performed a graded exercise test until voluntary exhaustion with pulmonary gas exchange analyses under normoxia. On the second, third, and fourth visits, participants engaged in HIIT sessions performed in normoxia or in two different normobaric hypoxic conditions. The imposed normobaric hypoxia condition delivered inspired air with an FIO_2_ equivalent to 0.13, which mimics the oxygen pressure at 3,600 m of altitude. During the HIIT sessions, heart rate (HR), blood lactate, rate of perceived exertion (RPE), peripheral artery oxygen saturation (SpO_2_, only during recovery phases), pulmonary gas exchange and peripheral muscle oxygenation (vastus lateralis) were monitored; during the recovery periods, arterial SpO_2_, blood lactate, and RPE were recorded.

### Hypoxia and normoxia conditions

The normobaric hypoxia air was delivered through a T-valve (Hans-Rudolph^®^, Kansas City, USA) attached to the mask that participants wore during the HIIT sessions. The hypoxia air was produced by a hypoxia generator (CAT-9 Air Unit-Everest Summit II™, Cardiff, USA) connected to an air storage system made of nylon balloons as detailed described by Norberto et al. (2024). This apparatus contains a set of valves to allow for the switch between the intake of ambient air and stored hypoxic air and was used in all three HIIT sessions. The participants could not visualize how the controlling valves were operating to ensure their blindness as to their FIO_2_ condition.

### The graded exercise test

On the first visit, participants ran on a motorized treadmill (Inbramed, Super ATL, Porto Alegre, Brazil) until voluntary exhaustion. The protocol consisted of a 5-minute warm-up at 7 km/h, followed by repeated increases in speed by 1 km/h every 2 min. In the case of exhaustion occurring in an incomplete stage, the V_MAX_ was calculated as the speed of the last completed stage plus 1 km/h multiplied by the fraction of time (in relation to 2-minute) spent on the incomplete stage.

### HIIT sessions

The HIIT sessions consisted of ten 1-minute bouts at 120% of V_MAX_ with 2 min of recovery. Before each HIIT session, participants sat for 2 min to obtain all baseline variables, which was followed by a 5-minute warm-up run at an individual low intensity (subjectively determined by the participant). These phases occurred regardless of the assigned FIO_2_ condition that would be imposed thereafter. At the end of the warm-up, participants were instructed to place their feet on the treadmill’s side rails until the speed was adjusted to reach the individually prescribed exercise intensity of 120% of V_MAX_. During this period that took one-minute, the participants began their exposure to the assigned experimental condition differing in FIO_2_. At the end of each one-minute effort, there was a 2-minute passive recovery (participants stood on the stopped treadmill or leaned on the treadmill support). Twenty seconds before starting a subsequent effort, participants waited with their feet on the treadmill’s side rails while the speed was set again to the prescribed intensity.

**Fig. 2:**
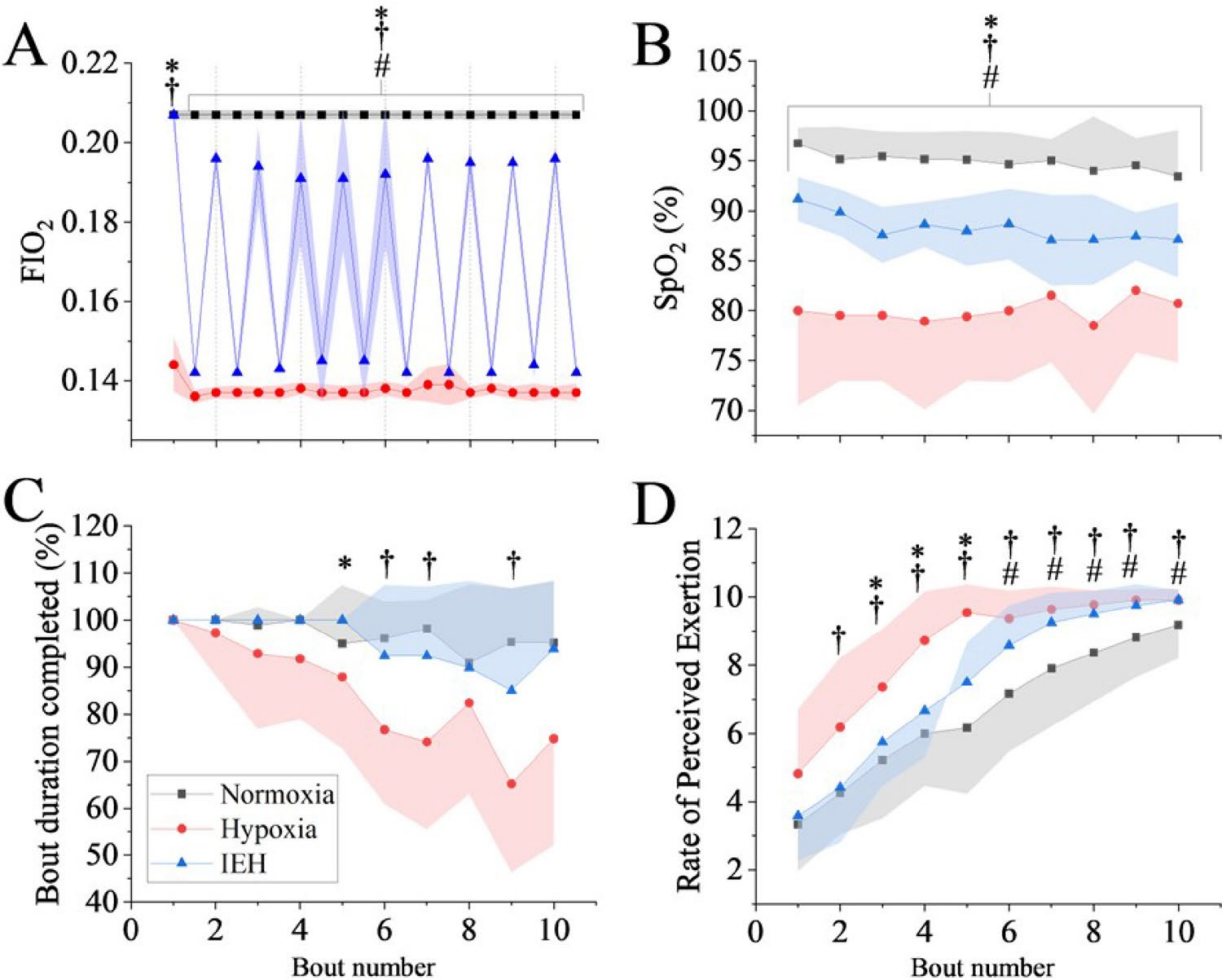
Hypoxia and its consequences for exercise tolerance during HIIT sessions. Data are mean ± standard deviation of values (*N* = 11) during each phase of the HIIT sessions. Differences across conditions were assessed using a generalized linear mixed model (GLM) with Bonferroni post-hoc. **A** The measured fraction of inspired oxygen (FIO_2_) during each effort and recovery. **B** Mean of peripheral arterial oxygen saturation (SpO_2_) during the recoveries. **C** Exercise tolerance as the percentage of each effort completed; **D**: The rate of perceived exertion assessed at the first minute into each recovery. * Difference between IEH and hypoxia; # difference between IEH and normoxia; † difference between hypoxia and normoxia

If participants failed to complete a given one-minute bout, their actual effort time was recorded and they were instructed to continue their attempt to complete the remaining bouts of the HIIT session protocol (the recovery duration after a bout failure was maintained at two-minutes). In this manner, regardless of the experimental conditions and being able to complete the entire bout of 60 s, all participants had 10 attempts to perform them. The averaging and the statistics of physiological data only included bouts, from 1 to 10, that were at least 90% completed (i.e., ≥ 54 s), except for data shown in Fig. 2. Each HIIT session was comprised of 10 attempts of efforts performed by 11 participants, generating a product of 110 expected bouts per exercise condition; from this total, the completion numbers were 63 (57%) in hypoxia, 100 (91%) in normoxia, and 99 (90%) in IEH recovery condition.

### Pulmonary gas exchange analyses

Oxygen uptake (V̇O_2_) and other respiratory variables were continuously measured breath-by-breath using the Quark CPET gas analyzer (Cosmed^®^ – Italy), calibrated immediately before use according to the manufacturer’s guidelines. For that purpose, a gas mixture with a known fraction of O_2_ (16%) and CO_2_ (5%) and a three-liter calibration syringe were employed to calibrate gas sensors and turbine. Participants continuously wore the standard face mask of the pulmonary gas exchange analyzer, but the T-valve was connected to the turbine only during HIIT sessions.

Initially, 5-s interval averages (i.e., 0–5, 6–10, 11–15) were calculated to analyze (Microsoft Excel) mean values of cardiorespiratory variables during the training session (VE, RER, PetCO_2_, PetO_2_, VO_2_/HR, VO_2_, and HR). Given the need for a more detailed analysis of VO_2_ kinetics, additional procedures were subsequently applied in R environment. All codes used for these procedures are available at GitHub (https://github.com/germanomputti/vo2-kinetics-ieh-training). Effort and recovery periods that contained six seconds or more without V̇O_2_ data, due to equipment error, were excluded from the final analyses (21/320 efforts and 21/320 recoveries). To standardize the number of data points in each trial, linear interpolation was performed using the “approx() function”, in order to obtain one data point per second. This procedure was required because data acquisition occurs on a breath-by-breath basis, and some consecutive breaths may be separated by more than one second. After interpolation, a moving average was applied to smooth the curves and reduce the influence of potential outliers on curve fittings. This was performed using the “rollmean() function” with the argument align = “right” and k = 7, meaning that each value corresponded to the mean of the previous seven seconds.

Using these processed data, the following variables were calculated: the total amount of oxygen consumed (i.e., the integral or area under the V̇O_2_-time curve), the amplitude of the response (maximum minus minimum V̇O_2_ values), and the time and absolute V̇O_2_ corresponding to 50% of the amplitude. Additionally, to investigate differences in the early and late phases of efforts and recoveries, the integral of the first 30 s and the last 30 s of each effort was calculated, as well as the integral of the first 30 s and the last 90 s of each recovery.

### Muscle oxygenation measurements

Muscle oxygenation (SmO_2_) was assessed by a NIRS device (PortaMon, Artinis^®^, Medical Systems BV, Zetten, Netherlands), placed 15 cm above the proximal border of the patella and 5 cm laterally on the vastus lateralis muscle (Osawa et al. 2017; Manchado-Gobatto et al. 2020; Marostegan et al. 2022). The device was securely attached and covered to block ambient light following shaving and cleaning of the skin with ethanol. The device features three light source transmitters (wavelengths of 760 and 850 nm) positioned at distances of 30, 35, and 40 mm from the receiver. The signals were processed using a 10th -order low-pass zero-phase Butterworth filter with a cutoff frequency of 0.1 Hz in the manufacter’s software (Oxysoft^®^, Artinis Medical System, Netherlands). Due to device features, the measured signal included the oxygenation status of both hemoglobin (Hb) and myoglobin (Mb), as most equipment are not capable of differentiating the contributions from the two hemi-containing protein (Barstow 2019). For simplicity, and to comply with the nomenclature from the manufacturer, the acronyms used to describe the oxygenation status of both heme proteins (Hb and Mb), we chose to employ only Hb acronym. The obtained SmO_2_ data were deoxyhemoglobin (HHb), oxyhemoglobin (O_2_Hb), total hemoglobin (tHb = O_2_Hb + HHb), and the tissue saturation index (TSI, calculated as O_2_Hb/tHb × 100). The shown O_2_Hb, HHb, and tHb values are the differences from baseline values measured before exercise began (Marostegan et al. 2022).

### Blood lactate

Asepsis of the ear lobe was performed with 70% alcohol before manual puncture with a sterile single-use stainless steel lancet (Wiltex). Then, 25 µL of blood were collected using pre-calibrated and heparinized capillary tubes. Blood samples were immediately transferred to 1.5mL tubes (Eppendorf^®^) containing 50 µL of 1% Sodium Fluoride (NaF) for subsequent lactate analysis using an electrochemical biochemical analyzer (Yellow Springs Instruments 2700, Ohio, USA).

### RPE and SpO_2_

The SpO_2_ was monitored by a pulse oximeter (G-Tech Portable, Rio de Janeiro, Brazil) every 10 s during the recovery phases of HIIT sessions. The pulse oximeter was positioned on the distal phalanx of the second finger of the participants. RPE was determined halfway into each recovery phase (i.e. at the first minute) of HIIT sessions by a 10-point scale (Foster et al. 2001).

### Estimation of the efficiency of pulmonary gas exchange

The alveolar-to-blood O_2_ diffusion gradient is a classical measure of pulmonary oxygen exchange efficiency. A non-invasive method, explored and validated by Prisk and West (2019) in hypoxic conditions, was used to estimate the difference between alveolar and arterial oxygen pressure. Accordingly, Hb-O_2_ binding affinity (i.e., the p50) was first calculated as a function of the end-tidal PCO_2_ (PetCO_2_, a proxy for arterial PCO_2_) with pH and temperature set at standard values, via Kelman’s equation (Dash and Bassingthwaighte 2010). SpO_2_ from pulse oximetry was used to generate the estimated arterial O_2_ pressure (ePaO_2_), but only in conditions exhibiting SpO_2_ < 95%, which is the range that yields good accuracy (Prisk and West 2019). For this reason, PaO_2_ during recovery in normoxia condition (where high SpO_2_ values are observed) was set at 105 mmHg (Prefaut et al. 2000) for all individuals rather than estimated from SpO_2_. Finally, end-tidal O_2_ pressure (PetO_2_) was taken as an estimation of alveolar O_2_ pressure, allowing for the calculation of PetO_2_ to *e*PaO_2_ difference. This is a non-invasive proxy of the classical alveolar gas equation from Riley’s concepts (Riley and Cournand 1949; Prisk and West 2019; West et al. 2020).

### Statistical analysis

Statistical analyses were performed in SPSS (IBM SPSS Statistics v20, New York, USA). Data normality was assessed using the Shapiro–Wilk test. Outcomes expressed as the average across all repetitions (Table 1, and left panel of Fig. 7) were compared using one-way repeated-measures ANOVA followed by Bonferroni post hoc tests. For outcomes not meeting parametric assumptions comparisons were performed using the Friedman test with Durbin–Conover post hoc correction.

**Fig. 3:**
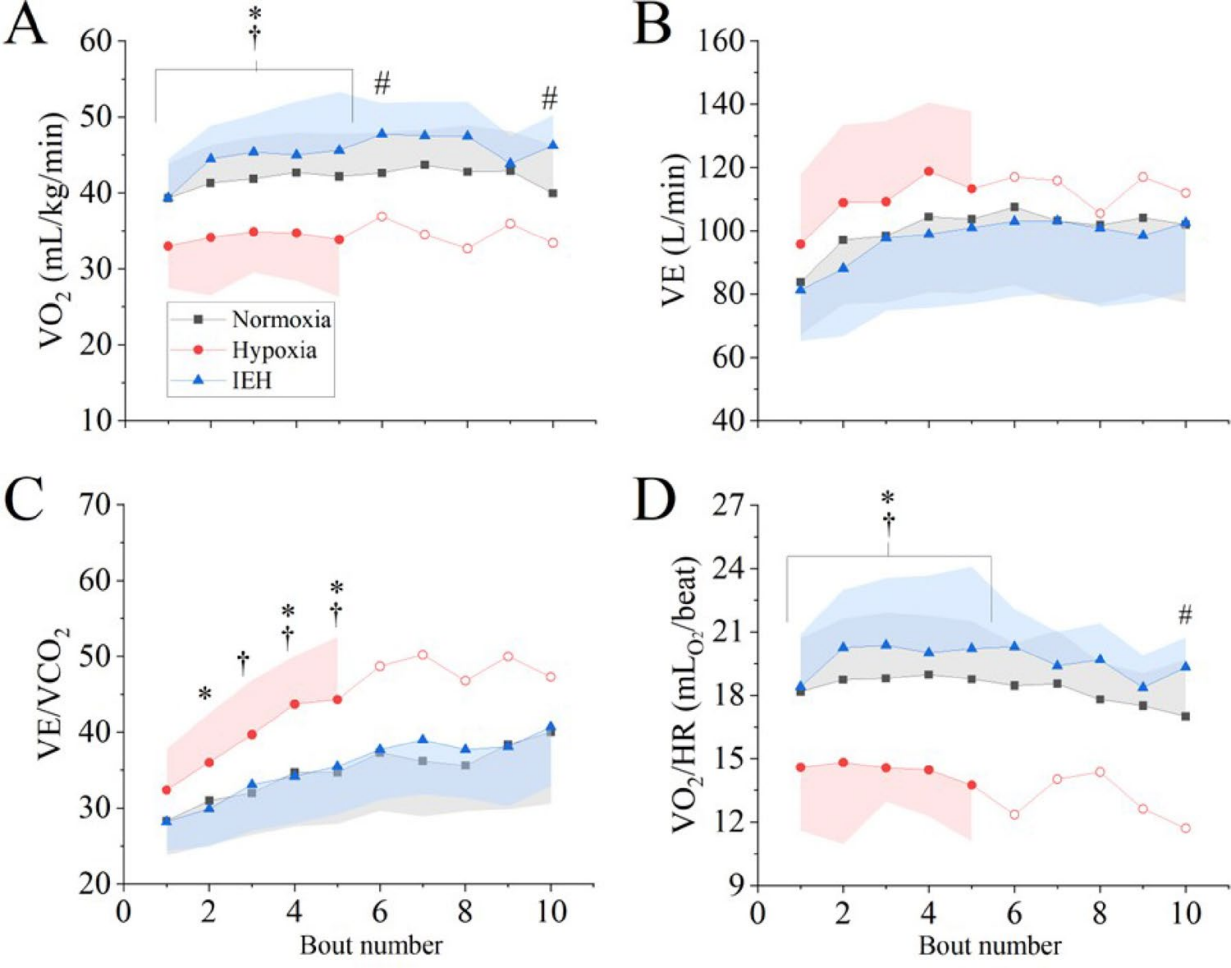
Selected cardiorespiratory variables during each of the 10 efforts of HIIT sessions. Data are mean ± standard deviation of values during each of the efforts lasting at least 54s. Differences across conditions were assessed using a generalized linear mixed model (GLM) with Bonferroni post-hoc. Data from the whole effort duration were averaged. **A** pulmonary V̇O_2_; **B** pulmonary ventilation; **C** the ventilatory equivalent of CO_2_ release; **D** O_2_ uptake pulse. Open circles represent descriptive measures unavailable for statistical analysis due to the number of participants included (less than half of the total n).* HR* heart rate. * Difference between IEH and hypoxia; # difference between IEH and normoxia; † difference between hypoxia and normoxia

Variables measured repeatedly across intervals (effort and recovery phases) were analyzed using mixed-effects generalized linear models (Figs. 2, 3, 5, 6, and 9) with subjects treated as random effects and condition and time as fixed effects. Depending on the distribution that best fit the data (Gaussian, Inverse Gaussian, or Gamma), selected according to the lowest Akaike Information Criterion (AIC), an identity link function was applied. Post hoc comparisons were adjusted using the Bonferroni method. Statistical significance was set at *p* < 0.05.

## Results

Moderate environmental hypoxia, with FIO_2_ of ~ 0.13, was elicited in both the continuous and intermittent manners, while FIO_2_ close to 0.21 characterized the normoxia condition. Figure 2 A shows FIO_2_ during each effort and recovery phases from the three experimental conditions during the HIIT sessions, evidencing that the hypoxia apparatus generated the three aimed experimental conditions: normoxia, hypoxia and IEH recovery. The effects of the HIIT sessions performed under those FIO_2_ conditions for SpO_2_ are depicted in Fig. 2B.

Both hypoxia and IEH recovery promoted significant decreases in SpO_2_ during the recovery phases (*p* < 0.05), compared to the control normoxic condition; the decreases in SpO_2_ was higher (*p* < 0.05) during hypoxia than on IEH recovery. HIIT sessions in both normoxia and under IEH recovery reached approximately 87% of the prescribed physical task (i.e., 10 efforts of 60 s), with no difference between these two conditions for the summed effort duration. However, the session of HIIT under hypoxia led to premature exhaustion, at around 49% of the prescribed task (Table 1). The median ± interquartile range for the duration of task completed were 600 ± 140 s, 338 ± 189 s and 600 ± 190 s, respectively, for normoxia, hypoxia and IEH conditions; there was only significant differences between hypoxia and the other two groups for summed effort duration (*p* < 0.001).

Because participants continued their 10 attempts to perform bouts of 60 s, even after a failure to complete a given one, the average duration of each bout is presented in Fig. 2C as the percentage of 60 s. These data add to the description of the work performed during the three experimental conditions, shown in Table 1, strengthening the analysis of the physical task completion during the HIIT sessions. Overall, the percentage of bout duration completed was significantly (*p* < 0.05) lower in hypoxia than in the other two conditions (Fig. 2C). RPE was assessed after each effort during HIIT sessions (Fig. 2D); in hypoxia, RPE were higher (*p* < 0.05) than in normoxia from bouts 2 to 10; IEH only generated higher (*p* < 0.05) RPE values compared to normoxia after the sixth bout.

Mean values for ventilatory and cardiometabolic variables from all efforts and recoveries are shown in Table 1. PetO_2_ was lower during efforts and recovery phases of HIIT sessions in the hypoxia and IEH recovery conditions (*p* < 0.05), compared to normoxia, which somehow mirror the imposed FIO_2_ shown in Fig. 2A. Blood lactate levels and heart rate during efforts and recoveries were not changed across the conditions of HIIT (Table 1). Compared to normoxia, mean V̇O_2_ during the 10 efforts was lower (*p* < 0.05) in hypoxia, but conversely higher (*p* < 0.05) during the IEH condition (Table 1); such a difference was only statistically significant at bouts 6 and 10, when each effort was analyzed independently (Fig. 3A).

Considering that heart rate did not change between conditions, oxygen pulse (i.e., V̇O_2_/HR) paralleled the changes in V̇O_2_ across experimental conditions (Table 1). There was evidence of hyperventilation during efforts and recoveries performed in hypoxia, as judged by lower PetCO_2_ during efforts of the HIIT session in the hypoxia, and during the recovery phases of the IEH recovery condition (Table 1). However, if the ventilatory equivalent of V̇CO_2_ (i.e., V_E_/V̇CO_2_) is taken as the index of hyperventilation, only the hypoxia condition led (*p* < 0.05) to hyperventilation during the efforts 3 to 5 (Fig. 3C).

**Table 1:** Overall average of cardiorespiratory variables during the efforts and recoveries of HIIT sessions in normoxia, hypoxia, and IEH

Variables	Normoxia (*n*=11)	Hypoxia (*n*=11)	IEH (*n*=11)
During the 10 efforts			
VE (L/min)	101.1 ± 22.2	98.5 ± 23.7	96.2 ± 20.3
RER	0.9 ± 0.1	1.1 ± 0.2	0.8 ± 0.1*#
PetCO_2_ (mmHg)	32.6 ± 6.7	28.4 ± 7.2*	32.4 ± 5.7#
PetO_2_ (mmHg)	107 ± 5.6	65 ± 7.7*	96 ± 8.3*#
V̇O_2_/HR (mL/beat)	18.3 ± 2.5	14.2 ± 2.6*	19.6 ± 2.6#
V̇O_2_ (mL/kg/min)	42.2 ± 4.8	33.6 ± 6.1*	45.1 ± 5.4#
HR (bpm)	158 ± 15	154 ± 22	159 ± 13
During the 10 recoveries			
Lactate (mmol/L)	9.9 ± 4.7	10.9 ± 3.3	10.8 ± 4.2
VE (L/min)	69.9 ± 20.2	71.8 ± 23.7	70.2 ± 25.1
RER	1.1 ± 0.1	1.2 ± 0.2	1.2 ± 0.2
PetCO_2_ (mmHg)	34.1 ± 7.2	30.1 ± 7.8*	30.5 ± 7.1
PetO_2_ (mmHg)	110 ± 5.3	64.5 ± 9.9*	67.1 ± 12.5*
V̇O_2_/HR (mL/beat)	12 ± 2.1	10.2 ± 2.8*	10.3 ± 3.1*
V̇O_2_ (mL/kg/min)	26.2 ± 4.9	23.2 ± 6.2	22.6 ± 6.2*
HR (bpm)	145 ± 17.7	145 ± 25	143 ± 31.1

Only participants completing 90% of the effort (54s) were included

Data are presented as mean ± standard deviation and were tested via ANOVA (with Bonferroni as a post-hoc).* IEH* Intermittent Hypoxia Interval Training,* FIO*_2_ Fraction of inspired O_2_,* SpO*_2_ peripheral arterial oxygen saturation,* V̇O*_2_ O_2_ consumption,* Rf* respiratory frequency,* VE* Ventilation,* HR *Heart rate,* RER* Respiratory Exchange Ratio quotient,* PetO*_2_ Partial pressure of end-tidal O_2_,* PetCO*_2_ Partial pressure of end-tidal* CO*_2_,* V̇O*_2_/*HR* O_2_ consumption and heart rate ratio

* different from normoxia; # different from hypoxia

**Fig. 4:**
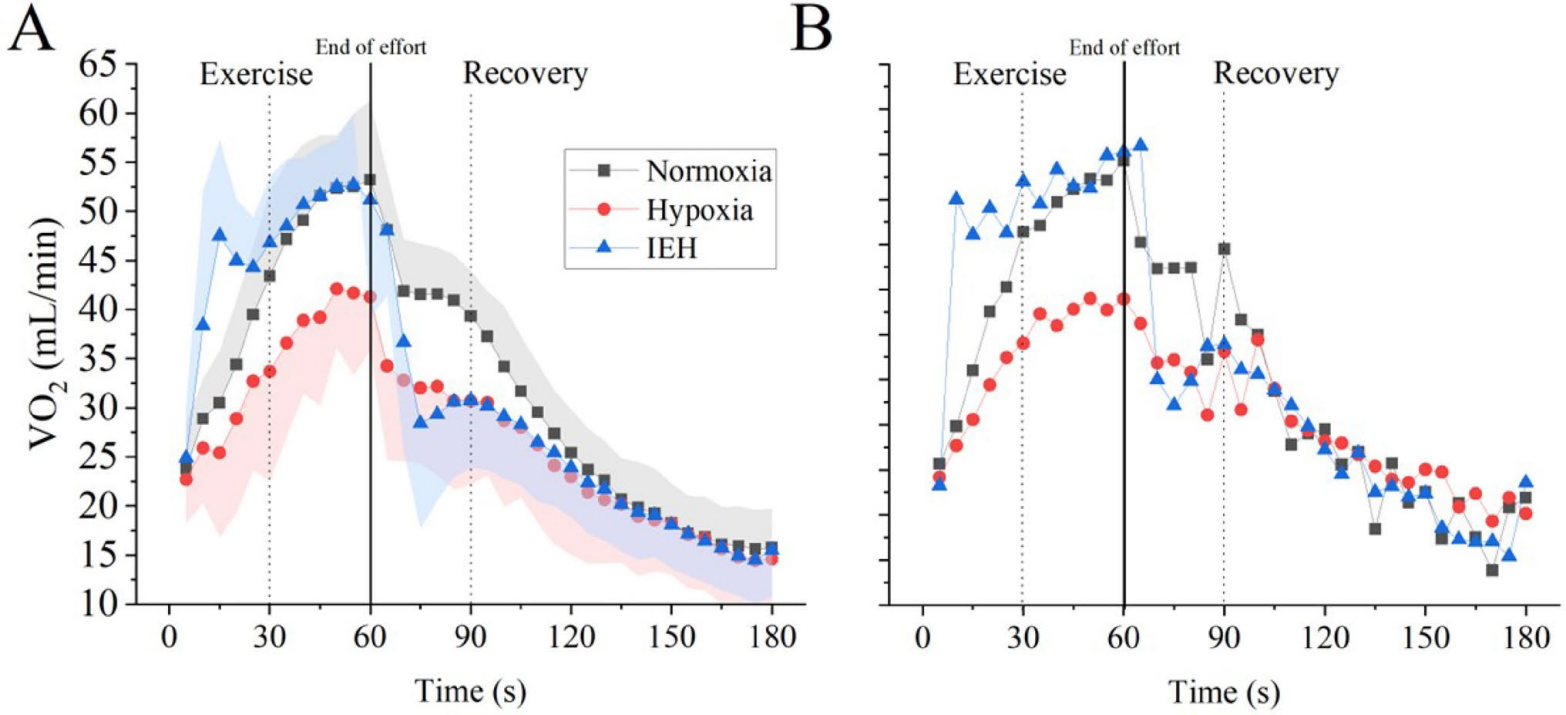
The time course of V̇O_2_ during efforts and recovery phases. **A** V̇O_2_ data during each effort and recovery phase were averaged over a 5-s window; then, the 10 efforts and recovery curves were averaged as mean ± standard deviation (shaded areas). **B** representative curves of V̇O_2_ time curses from subject 4 (smoothed data of the 6^th^ effort plus the following recovery phase). The end of the efforts is at the 60 s vertical line, which also marked the transition to the recovery phase that lasted for further 120 s

Individual V̇O_2_ data were averaged in 5-s windows and the mean time-course across the 10 efforts and recoveries was calculated to illustrate the overall kinetic profiles for each condition (Fig. 4A). Qualitatively, these averaged curves suggested condition-dependent differences in the V̇O_2_ response. To better visualize the underlying kinetic pattern that motivated subsequent analyses, an example of an individual smoothed V̇O_2_ curve is also presented (Fig. 4B). As detailed in the Methods section, qualitative kinetic parameters were derived from these smoothed curves to characterize the temporal features of the V̇O_2_ response.

**Fig. 5:**
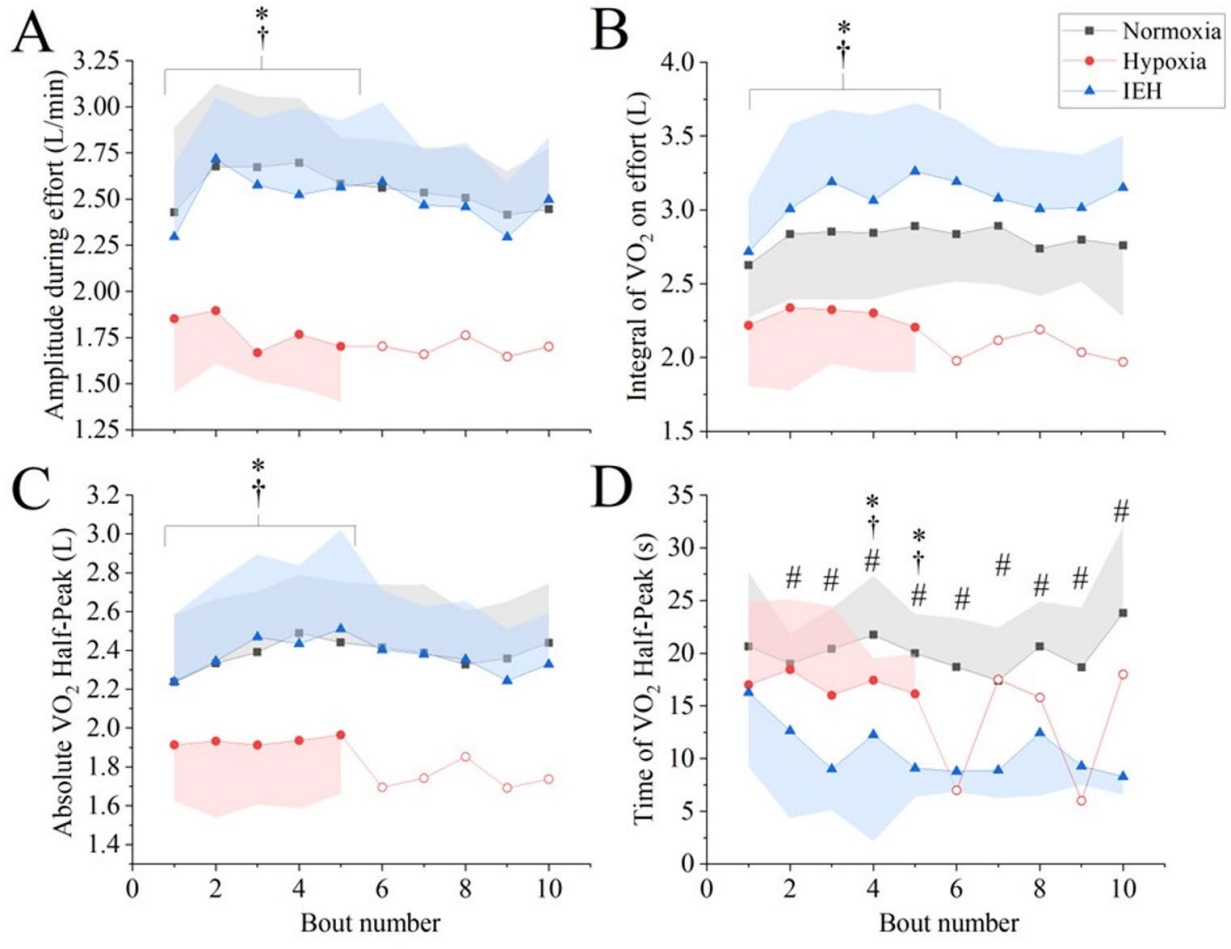
Total oxygen uptake during each effort and kinetic parameters to describe the increase curves. Data points are mean ± standard deviation. Differences across conditions were assessed using a generalized linear mixed model (GLM) with Bonferroni post-hoc. **A** the amplitude of the increased response over the 60s effort. **B** total oxygen uptake during effort was computed as the integral of the V̇O_2_ curve. **C** V̇O_2_ value associated with half of the peak amplitude during each effort. ** D**: time spent to reach half of the peak amplitude during each effort. Only participants completing 90% of the effort (54s) were included. Open circles represent descriptive measures unavailable for statistical analysis due to the number of participants included (less than half of the total n). * Difference between IEH and hypoxia; # difference between IEH and normoxia; † difference between hypoxia and normoxia

For each effort and recovery phase, V̇O_2_ smoothed curves were analyzed and the following parameters were determined: (i) integral (the area under the curve); (ii) the amplitude of the response; (iii) the time and absolute V̇O_2_ at the half of the amplitude. The conditions of IEH recovery and normoxia showed higher V̇O_2_ amplitude, V̇O_2_ integral, and V̇O_2_ at half-peak than hypoxia during the first five efforts (Fig. 5A); latter efforts under hypoxia were not statistically analyzed because participants failed to accomplish the whole bout duration. A shorter time to reach V̇O_2_ half-peak was observed during efforts 2 to 10 in the IEH recovery condition, compared to normoxia; for this variable, IEH also presented a shorter time than hypoxia during the 4th and 5th efforts (Fig. 5).

**Fig. 6:**
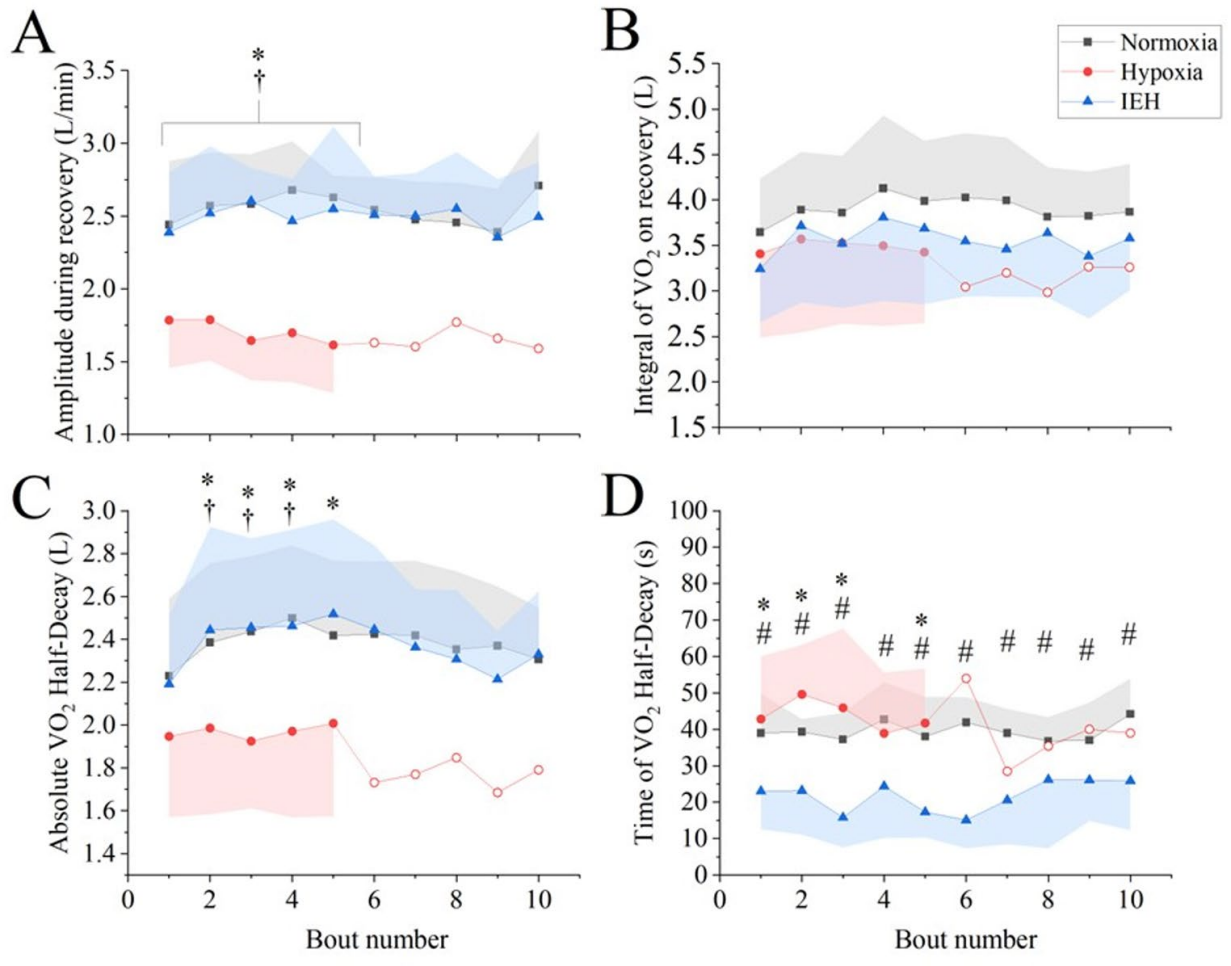
Total oxygen uptake during each recovery and kinetic parameters to describe the decay curves. Data points are mean ± standard deviation. Differences across conditions were assessed using a generalized linear mixed model (GLM) with Bonferroni post-hoc. ** A** the amplitude of the decay response over the 2-minute recovery. ** B** total oxygen uptake during recovery was computed as the integral of the V̇O_2_ curve. ** C** V̇O_2_ value associated with half of the decay amplitude during each recovery. ** D**: time spent to reach half of the decay amplitude during each recovery. Only participants completing 90% of the effort (54s) were included. Open circles represent descriptive measures unavailable for statistical analysis due to the number of participants included (less than half of the total n). * Difference between IEH and hypoxia; # difference between IEH and normoxia; † difference between hypoxia and normoxia

Because data in Fig. 3 suggested that the differences between conditions were mostly in the first 30 s, we also analyzed V̇O_2_ × time integral in the first and second half of the 60 s efforts (Fig. 6A) and the first 30s and last 90s of recoveries (Fig. 6B). Indeed, such an individual analysis of curves showed that the IEH recovery led to higher V̇O_2_ integral only during the first 30 s of effort, compared to other experimental conditions. On the other hand, hypoxia only promoted a lower V̇O_2_ integral than the other conditions during the final 30 s of efforts (Fig. 6A). During recovery, lower V̇O_2_ integrals were observed in hypoxia and IEH recovery conditions compared to normoxia, both in the first 30 s and for the total recovery period of 120 s (Fig. 6B).

**Fig. 7:**
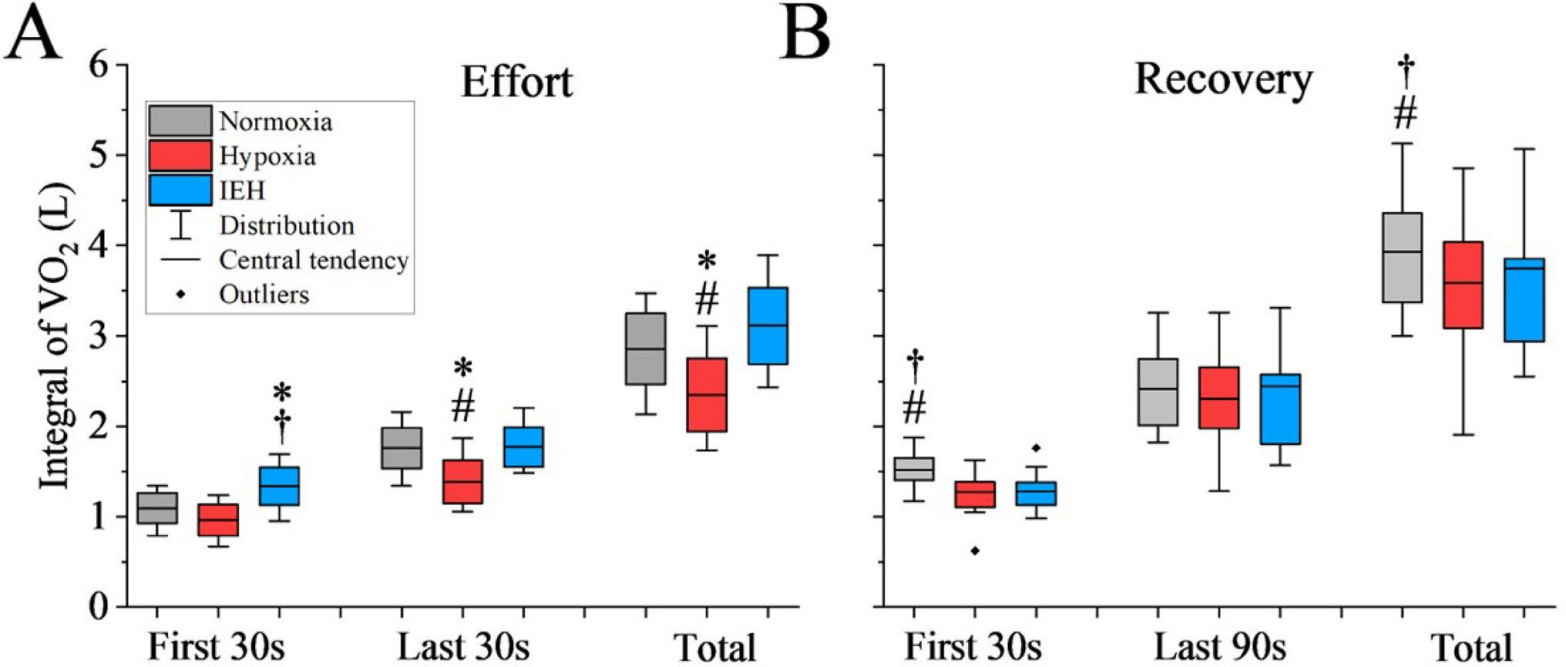
Analysis of the O_2_ uptake integral across different fractions of the effort and recovery. The effort panel (left side) is presented as mean ± standard deviation (Repeated measures ANOVA analysis), while the recovery panel (right side) is shown as the median ± interquartile range (Friedman analysis). Only participants completing 90% of the effort (54s) were included. * indicates a significant difference from normoxia; † indicates a significant difference from hypoxia; # indicates a significant difference from IEH

Because data in Fig. 4 suggested that the differences between conditions were mostly in the first 30 s, a V̇O_2_ kinetics’ analysis was also performed in the first and second half of the 60 s efforts (Fig. 7A). Indeed, such an individual analysis of curves showed that the IEH recovery led to higher V̇O_2_ integral only during the first 30 s of effort, compared to other experimental conditions. On the other hand, hypoxia only promoted a lower V̇O_2_ integral than the other conditions during the final 30 s of efforts (Fig. 7A). During recovery, lower V̇O_2_ integrals were observed in hypoxia and IEH recovery conditions compared to normoxia, both in the first 30 s and for the total recovery period of 120 s (Fig. 7A). The parameters of the V̇O_2_ curve analysis for each recovery phase are shown in Fig. 7.

**Fig. 8:**
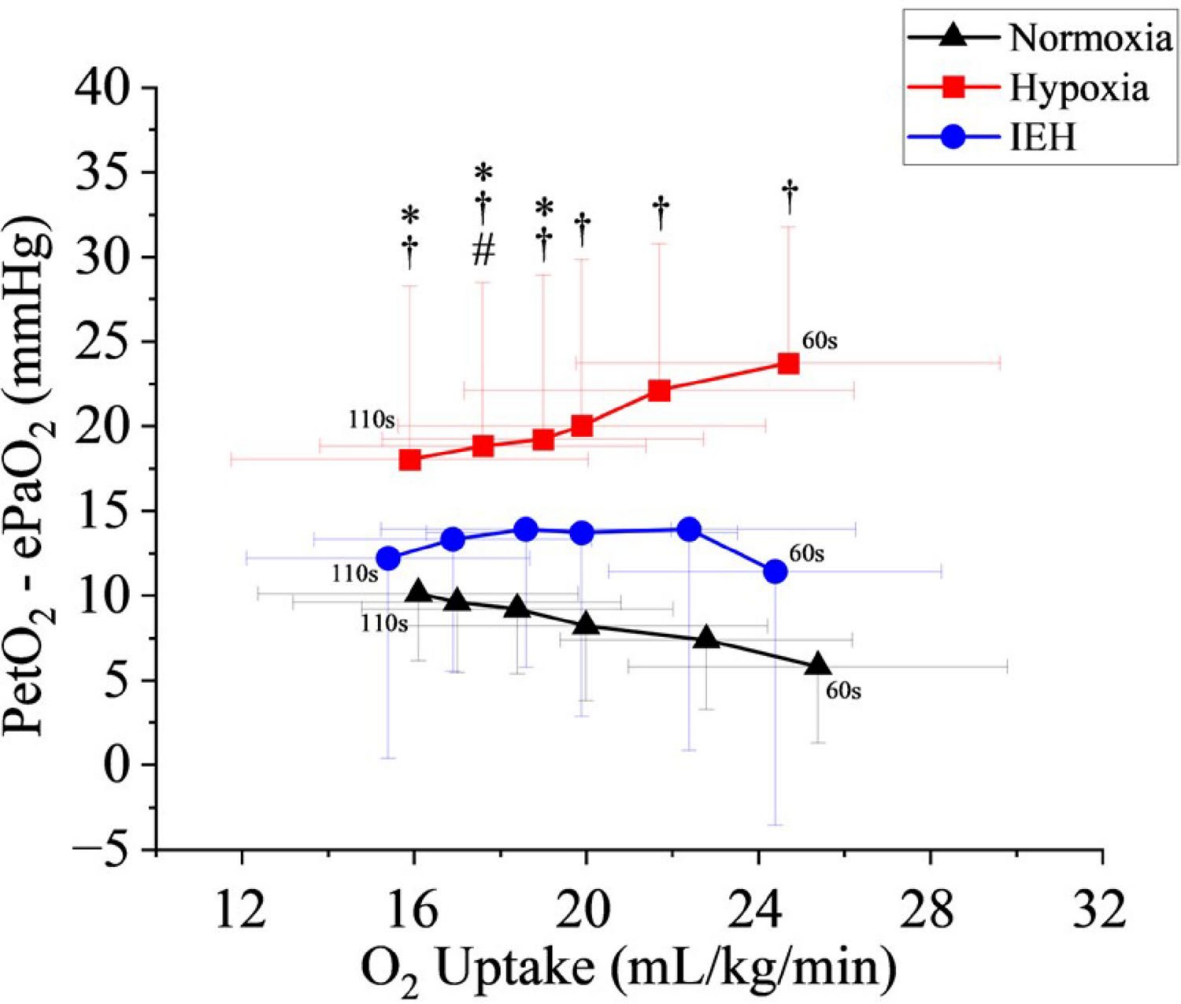
The efficiency of oxygen transport and utilization in relation to the alveolar-arterial oxygen gradient, as represented by the relationship between V̇O_2_ and PetO_2_-ePaO_2_ difference. Data are presented as mean ± standard deviation. Only participants completing 90% of the effort (54s) were included in this analysis. The data points were obtained from a time frame of the recovery phases (i.e., from 60 to 110 s). The variable depicted in y-axis is a known index (see Material and Methods) of the limitation for alveolar to capillary O_2_ diffusion* Difference between IEH and hypoxia; # difference between IEH and normoxia; † difference between hypoxia and normoxia

The estimated index of alveolar O_2_ diffusion limitation to arterial blood, the PetO_2_-ePaO_2_ plotted against V̇O_2_, was higher in hypoxia than in normoxia during the recovery phase (Fig. 8). IEH condition showed a lower PetO_2_-ePaO_2_ compared to hypoxia at the 60-, 70-, and 80-s of recovery. For this variable, higher values (*p* < 0.05) were observed in the IEH condition for last three data points representing the end of the recovery period, compared to normoxia (Fig. 8).

**Fig. 9:**
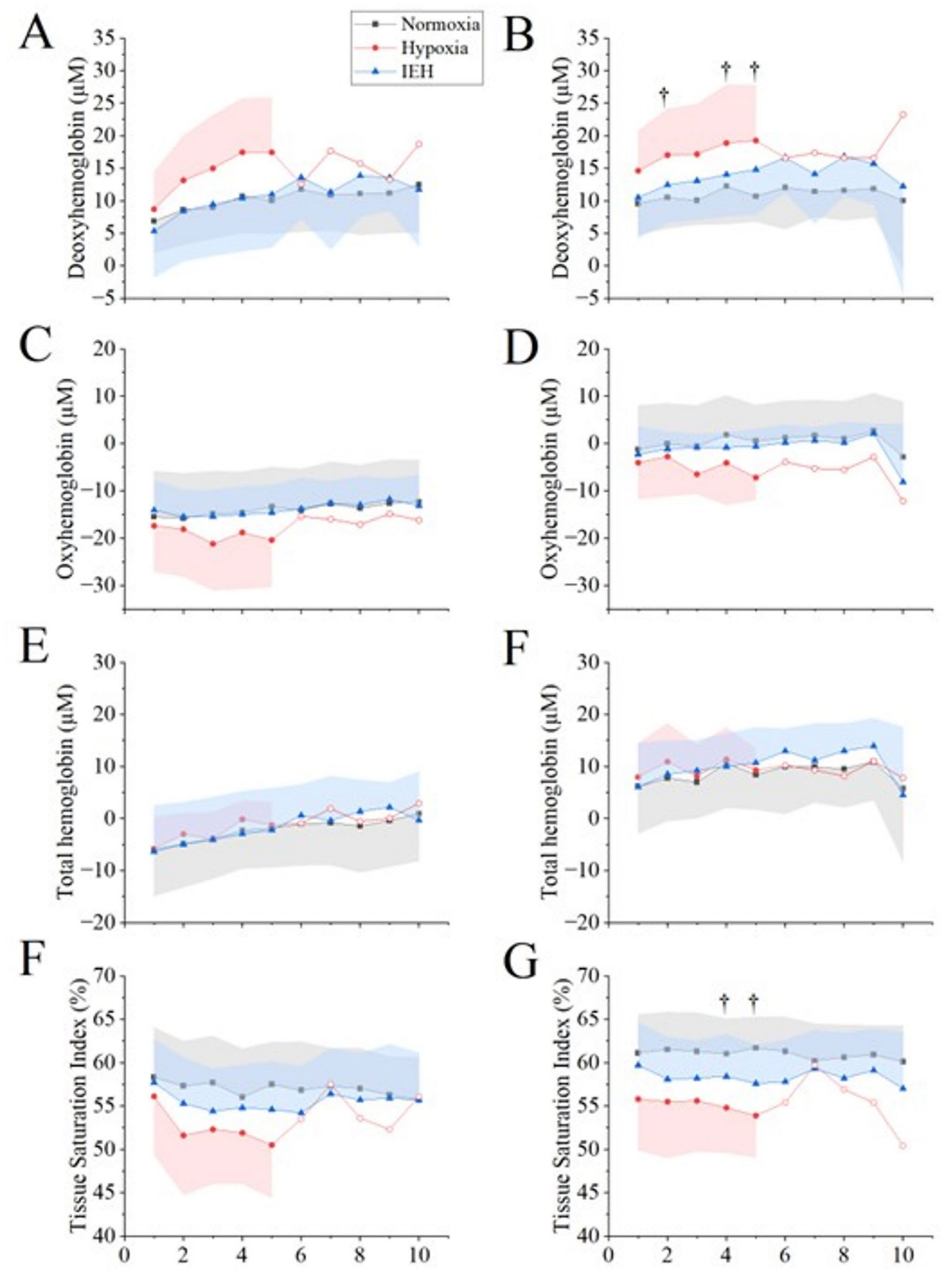
Analysis of the muscle oxygenation variables during effort and recovery.** A** Deoxyhemoglobin during efforts; **B** Deoxyhemoglobin during recoveries; **C** Oxyhemoglobin during efforts; **D** Oxyhemoglobin during recoveries; **E** Total hemoglobin during efforts;** F** Total hemoglobin during recoveries; **G** Tissue saturation index during efforts; **H** Tissue saturation index during recoveries. Data are shown as mean ± standard deviation. Data of HHb, O_2_Hb and tHb represent changes relative to the rest baseline (Δ), whereas TSI (%) reflects absolute saturation values. Squares in black, circles in red and triangles in blue, represent the conditions of normoxia, hypoxia and IEH, respectively. Only participants completing 90% of the effort (54s) were included. Open circles represent descriptive measures unavailable for statistical analysis due to the number of participants included (less than half of the total n). † - difference between normoxia and hypoxia

Regarding the average peripheral O_2_ delivery and extraction, NIRS data from the vastus lateralis indicated that hypoxia elicited a significantly lower TSI than normoxia during the efforts (52.3 ± 6.1 and 55.4 ± 4.7 respectively). During the recovery phases, both IEH and hypoxia showed reduced TSI compared to normoxia (56.8 ± 4.7, 55.2 ± 5.4, and 59.6 ± 4.1 respectively). When each bout and recovery was analyzed individually, hypoxia presented lower TSI than normoxia on the 4th and 5th recoveries (Fig. 9G) and also exhibited higher HHb than normoxia during the 2nd, 4th, and 5th recovery periods (Fig. 9B).

## Discussion

This study aimed to investigate whether applying hypoxia during IEH would increase systemic V̇O_2_ without compromising exercise tolerance and mitigate the muscle oxygenation reduction compared to continuous hypoxia. Our findings support these hypotheses, showing that IEH enhances oxygen consumption and attenuates muscle deoxygenation, while maintaining exercise intensity. This assertion is clear depicted by the mean V̇O_2_ curves in Fig. 4, which indicate a faster V̇O_2_ adjustment during the first 25-s of HIIT efforts in the IEH recovery condition compared to normoxia.

The quantitative analysis of these curves, based on the binning 60-s efforts into two halves (Fig. 5), further supports this finding. Specifically, aerobic metabolism was enhanced during the first 30-s of efforts following hypoxic recovery. Notably, traditional curve-fitting approaches to describe V̇O_2_ kinetics, such mono- and bi-exponential adjustment (Ozkaya et al. 2023), did not appear to adequately capture the observed curve shapes (Fig. 4). It has been demonstrated that V̇O_2_ kinetics during the transition from rest to exercise (at intensities above the lactate threshold) is accelerated when O_2_ delivery to the skeletal muscle is increased (Macdonald et al. 1997). Compensatory vasodilatation, along with elevated pulmonary and muscle blood flow, are expected acute responses to hypoxia exposure (Meng et al. 2019; Burtscher et al. 2022) that help mitigate decreases in O_2_ transport under these conditions. In this regard, the relationship between the alveolar-to-blood O_2_ diffusion gradient (PetO_2_-ePaO_2_) and V̇O_2_ during recovery indicated that both hypoxia and IEH conditions promoted compensatory adaptations-primarily at the level of circulation/perfusion-to sustain V̇O_2_ (Fig. 8).

The utility of the PetO_2_-ePaO_2_ as a valid index of pulmonary diffusion limitation to O_2_ transport in hypoxemia, as observed in the present study due to the lower FIO_2_ and SpO_2_, has been previously demonstrated (Prisk and West 2019; West et al. 2020). Pulmonary gas exchange equations predict that impaired alveolar-capillary O_2_ transfer must be offset by increased blood flow in hypoxia to preserve O_2_ uptake (Piiper and Scheid 1981; Beretta et al. 2019). Presumably, blood flow could be enhanced during recoveries in hypoxia, leading to an overshoot in O_2_ delivery and utilization (Fig. 4). Importantly, this overshoot is favored by the abrupt transition from hypoxia during recovery to normoxia at exercise onset, a condition unique to the IEH protocol and not present in continuous hypoxia. This phenomenon, which reflects an excess of V̇O_2_ above the expected value during transitions from rest to exercise, has been reported under other conditions that similarly enhance determinants of skeletal muscle O_2_ utilization (Koppo et al. 2004; Figueira et al. 2009).

A key consideration regarding the IEH model is the effect of recovering in hypoxia on aerobic metabolism and exercise tolerance. A reasonable concern is that hypoxic recovery could partially suppress aerobic metabolism, impairing creatine phosphate resynthesis and, consequently, limiting performance in subsequent high-intensity efforts. However, the present data (Table 1 and Fig. 2C) and recent publications (de Carvalho et al. 2023; Foresti et al. 2023; Li et al. 2024) indicate that exercise tolerance may not be compromised in HIIT sessions with IEH recovery, despite O_2_ uptake during the recovery being nearly 15% lower than in normoxia (Table 1). The classical mechanism of creatine phosphate resynthesis established the aerobic metabolism as the source of ATP driving the creatine kinase-catalyzed reaction (Sahlin et al. 1979). While this mechanism remains undisputed here, it appears that the priming of oxidative metabolism for subsequent efforts offsets potential impairments in muscle performance due to hypoxic recovery.

Concerning perceptual responses, the RPE, which typically correlates with fatigue and exercise tolerance (Crewe et al. 2008), were higher after efforts 6 to 10 of the HIIT session in the IEH recovery condition compared to normoxia. Thus, it remains to be determined whether the IEH recovery does not affect exercise tolerance in other intermittent exercise protocols, particularly those involving all-out efforts. The present discussion has not focused on the more conventional hypoxia condition- continuous hypoxia throughout the HIIT session-which served as a positive control. However, this condition clearly induced physiologically meaningful hypoxemia, suppressed aerobic metabolism, increased RPE, and substantially decreased exercise performance (Table 1; Fig. 2).

Lower exercise tolerance in hypoxia is traditionally attributed to increased metabolic stress resulting from reduced arterial oxygen content (CaO_2_) and systemic/lower limb O_2_ delivery (Calbet et al. 2003). As a consequence of a Pasteur-like effect, anaerobic energy metabolism is typically increased in a compensatory manner during moderate or heavy exercise under hypoxia (Ibanez et al. 1993; Scott et al. 2016). However, in this study, blood lactate levels did not differ between normoxia and the two hypoxic conditions during HIIT at supramaximal intensity (Table 1). This finding aligns with our previous works (de Carvalho et al. 2023; Foresti et al. 2023) and an elegant study by Calbet et al. (2003), which reported that lactate levels in femoral blood — a more reliable indicator of anaerobic energy yield in active muscles — remained similar between normoxia and hypoxia during maximal-intensity cycling (Calbet et al. 2003).

The evaluation of muscle oxygenation using NIRS provided additional insights into muscle oxygenation status under different experimental conditions (Fig. 9). As expected, hypoxia resulted in slightly lower muscle oxygenation (i.e., lower tissue saturation index [TSI]) during both efforts and recoveries phases compared to normoxia. In contrast, IEH recovery only led to lower TSI during the recovery phases. No significant changes were observed in other NIRS variables, such as total hemoglobin (tHb) under hypoxic conditions, which has also been found in previous studies on exercise in hypoxia (Billaut and Buchheit 2013; Chacaroun et al. 2019). However, caution is warranted when comparing NIRS-derived data, as device specifications, exercise recovery duration (Raberin et al. 2023), and intensity (Kime et al. 2013) can influence the measured muscle oxygenation responses. Nonetheless, data in Fig. 2 evidenced that the IEH recovery condition decreased oxygenation at the active muscle level, potentially serving as an additional stimulus for peripheral response. In this regard, two studies have reported that training programs that incorporating IEH recovery sessions resulted in superior performance-related gains (Roels et al. 2005; Putti et al. 2024).

In conclusion, this study shows that applying IEH increases systemic V̇O_2_ without impairing exercise tolerance. Compared to continuous hypoxia, IEH also mitigates muscle deoxygenation, suggesting improved oxygen delivery and redistribution. These findings highlight IEH as a practical and effective strategy to integrate hypoxic stimuli without reducing exercise intensity. Further studies are needed to investigate the chronic adaptations and performance benefits of this approach. 

This study received funding from the São Paulo Research Foundation through Grants FAPESP: 2023/02728-3, 2021/02403-1, 2020/11946-6, 2019/20930-9, 2019/20894-2, and 2019/10666-2. The authors declare no conflicts of interest. All procedures were approved by the University Ethics Committee (protocol number: 32220020.0.0000.5659) and conducted in accordance with the Declaration of Helsinki. The authors express their gratitude to the Laboratory of Applied Sport Physiology (LAFAE), the Group of Studies on Physiology and Exercise (Gecifex), as well as all collaborators and volunteers who contributed to this research.
